# Antibiotics and probiotics on hepatic venous pressure gradient in cirrhosis: A systematic review and a meta-analysis

**DOI:** 10.1371/journal.pone.0273231

**Published:** 2022-08-30

**Authors:** Haonan Zhang, Jian Gao

**Affiliations:** 1 Second Clinical College, Chongqing Medical University, Chongqing, China; 2 Department of Gastroenterology, The Second Affiliated Hospital of Chongqing Medical University, Chongqing, China; Public Library of Science, UNITED KINGDOM

## Abstract

**Background:**

Modulation of the gut microbiome could favorably alter the hepatic venous pressure gradient (HVPG) in cirrhosis and portal hypertension (PH).

**Aim:**

This meta-analysis was to evaluate the effects of microbiome-targeted therapies (MTTs) on HVPG in persons with cirrhosis and PH.

**Methods:**

PubMed, The Cochrane Library, Embase, Web of Science and Scopus were searched for randomized clinical trials (RCTs) analyzing the effects on HVPG in people with cirrhosis who received MTTs. Clinical outcomes were pooled using RevMan5.3 software. A trial sequential analysis was applied to calculate the required information size and evaluate the credibility of the meta-analysis results.

**Results:**

A total of six studies were included. MTTs were associated with a reduction of 1.22 mm Hg in HVPG (95% CI: -2.31, -0.14 mmHg, P = 0.03). Subgroup analysis showed a greater reduction with longer duration (-1.88 mmHg;95% CI: -3.23, -0.53; P = 0.006). In the trial sequential analysis of HVPG reduction, the cumulative Z curve crossed the traditional significance boundary without the achievement of required information size (330).

**Conclusions:**

MTTs may be associated with a reduction in HVPG in patients with cirrhosis and PH. Microbiome-targeted therapies merit additional large-sample studies to define the efficacy of HVPG.

**Systematic review registration:**

PROSPERO 2020: CRD4202021609.

## Introduction

Portal hypertension (PH) is a typical syndrome of cirrhosis that may result in complications such as ascites, spontaneous bacterial peritonitis, gastroesophageal variceal bleeding and other conditions [1]. Even with appropriate management, the mortality is approximately 15% to 20% among fatal complications [2]. The correlation between the increase in hepatic venous pressure gradient (HVPG) and the occurrence of PH has been well documented. HVPG>5 mmHg indicates portal hypertension. Clinically significant portal hypertension (CSPH) was defined by a HVPG ≥10 mmHg [3, 4]. An observation has shown that with a reduction in HVPG>20% from baseline or to levels below 12 mmHg, PH-related complications and mortality were significantly reduced [5, 6]. Nonselective beta-blockers (NSBBs) are the available medications for PH and show a sufficient decrease in HVPG [7, 8]. However, only a minority of patients (approximately 30% to 50%) exhibit a meaningful clinical response during NSBB therapy [8]. In addition, approximately 15% of patients have absolute or relative contraindications to therapy [9]. As a result, novel therapeutic approaches are imperative.

Gut-liver axis emphasizes the close relationship between the gut and the liver. Bacterial translocation (BT) transmits bacteria or their products from the gastrointestinal tract to normally sterile tissues [10, 11], occurring in 25% to 30% of cirrhosis patients [12]. BT results in the release of proinflammatory cytokines, such as lipopolysaccharide binding protein (LBP), tumor necrosis factor-α (TNF-α), interleukin-6 (IL-6) and nitric oxide (NO). Proinflammatory environment finally promotes hepatocyte injury and fibrosis [13], exacerbating the hyperdynamic circulatory state and increasing hepatic vascular resistance [14–16]. As such, there is a potential for intestinal flora to be a target in PH.

Microbiome-targeted therapies (MTTs) can be divided into four categories, namely antibiotics, prebiotics, probiotics and fecal microbiota transplantation (FMT). Antibiotics such as rifaximin and norfloxacin selectively decontaminate the intestines, which could lead to HVPG changes [17, 18]. Probiotics could alter the make-up of the intestinal microbiome to decrease endotoxemia [19]. In this way, probiotic therapy might have a beneficial effect in patients with cirrhosis and PH. Given the limitation of a single type of MTT and the conflicting results, a recent meta-analysis points to a need for additional investigations.

Therefore, we aimed to conduct a meta-analysis of randomized controlled trials (RCTs) to examine the influence of microbiome-targeted therapies (MTTs) on hepatic portal venous pressure in cirrhosis with PH.

## Method

We established a protocol for the review, which was registered with PROSPERO prior to commencing the study. (CRD42020216092)PRISMA checklist is provided in S1 File.

### Study selection

Randomized controlled trials (RCTs) comparing MTTs against regular medication use, placebo, or a blank control in patients with cirrhosis and PH qualified for inclusion. MTTs were defined as follows: antibiotics, prebiotics, probiotics and fecal microbiota transplantation (FMT). Eligible RCTs met the following criteria: 1) patients were diagnosed with cirrhosis and PH; The diagnosis of cirrhosis was either liver biopsy proven or clinically suspected based on image studies and biochemical criteria. Portal hypertension was diagnosis by HVPG ≥10mm Hg or endoscopically documented large esophageal varices. 2) had an intervention group receiving MTTs; 3) had a control group receiving placebo or control medication; and 4) HVPG was evaluated before and after therapy in both the MTT and control arms. The exclusion criteria were as follows: 1) unpublished studies or studies available in abstract or letter form only; 2) HVPG measurement data were lost at baseline or endpoint, and 3) an absence of control groups.

### Identification and selection of studies

We searched five electronic databases: PubMed, The Cochrane Library, Embase, Web of Science and Scopus. We used search terms such as (“cirrhosis”) and (“Portal hypertension”) and (“Antibiotics” or “Probiotics” or “Prebiotics” or “Fecal Bacteria Transplant”). All database searches were based on the combination of subject words and free words. Two reviewers independently investigated the titles and abstracts of studies and excluded irrelevant trials. Then each potential study was examined by two reviewers through full-text reading to assess whether the trial met the inclusion criteria. In cases of disagreement, the two reviewers reached consensus after discussion. The full electronic search strategy was list in the S2 File.

### Data extraction and study appraisal

A predesigned, standardized form was used to extract data from each included study. The information included: 1) study characteristics, including publication date, country, first author, inclusion or exclusion criteria and related details; 2) patient characteristics, such as number of patients, age, gender distribution, Child-Pugh and MELD score, and etiology of cirrhosis; 3) interventions (type and dose of MTT and duration); and 4) clinical outcomes, as previously chosen. For all articles with missing details, the corresponding authors were contacted via email to request the information. If it was not possible to obtain the data, the study was excluded from the data synthesis. We assessed all the included studies for methodological quality with the use of the Cochrane Risk of Bias tool form with the following six aspects: random sequence generation, allocation concealment, blinding, incomplete outcome data, selective reporting of results and other biases.

### Outcomes

The main outcome was HVPG reduction in patients treated with MTT compared with patients receiving control treatment. The additional outcomes were the plasma concentrations of BT-related markers and inflammatory cytokines. We reviewed all the included studies and chose LBP, IL-6, TNF-α and NO as the second outcomes.

### Synthesis and statistical analysis

This meta-analysis was performed by RevMan5.3 software. All continuous variables are expressed as the means ± standard deviations (SDs). When the original article did not report the mean or SD, we estimated them by the equation using the median, quartile and range. The continuous effect amounts were reported as the mean differences (MDs) and 95% confidence intervals (CIs). When different scales or large differences between numbers were used in each trial, standardized mean differences (SMDs) and 95% confidence intervals (CIs) were chosen as the combined statistics. Heterogeneity of results between studies was evaluated by chi-squares and I^2^ values, either chi-squares test P < 0.10 or I^2^ values > 50% indicated heterogeneity, and meta-analysis was performed using a random effect. At the same time, subgroup analyses were also performed to explore the potential source of heterogeneity; otherwise, a fixed effect model was utilized.

A trial sequential analysis (TSA, version 0.9.5.10 beta) was performed to calculate the required information size (RIS) and the trial sequential monitoring boundaries. In the TSA analysis, the probabilities of a type I error (α = 0.05) and type II error (β = 0.20) were used to calculate the RIS. The relation between the cumulative Z curve and the trial sequential monitoring boundary shows the credibility of the results.

## Results

### Included studies

**Fig 1:**
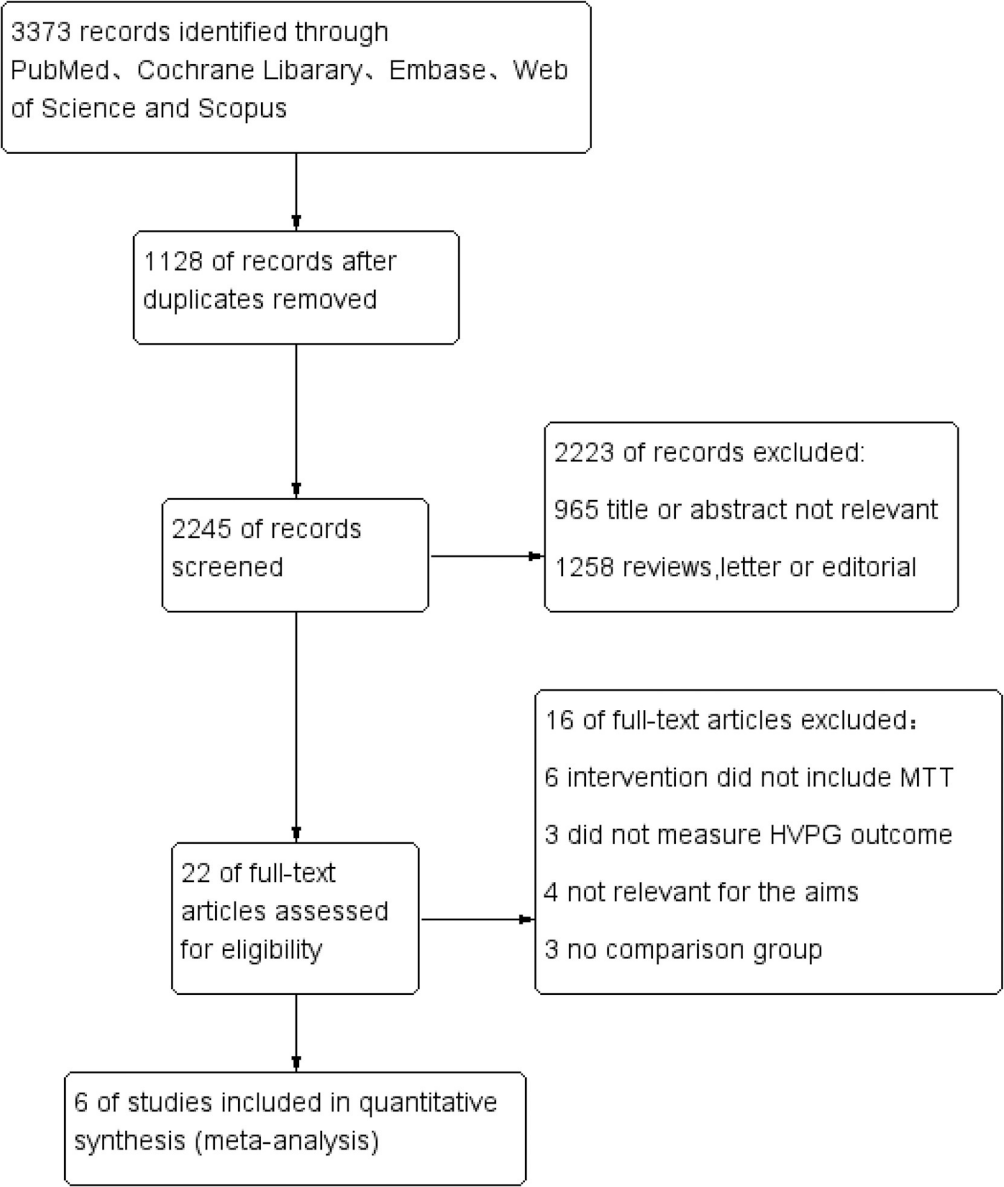
Flow chart of the screening process.

Fig 1 shows the study selection process. We identified 3373 records according to the predesigned search strategy, of which 1128 were duplicates. Twenty-two potentially relevant manuscripts were reviewed in full-text following title and abstract screening. After full-text reviews, a total of 6 documents [20–25] met the eligibility criteria and were included in the present review. Table 1 summarizes the characteristics of the included studies.

**Table 1:** The characteristics of the included studies.

Author	Year	Country	No.of patients	NSBB Use%	Inclusion Criteria
Albillos [20]	2003	Spain	18	NR	Cirrhosis + GEV^a^
Kemp [23]	2009	Australia	16	56.3	Cirrhosis + HVPG^b^ ≥ 12 mmHg
Jayakumar [22]	2013	Canada	15	NR	Cirrhosis + HVPG ≥ 10 mmHg+ CP^c^ class B or C
Gupta [21]	2013	India	94	100	Cirrhosis + large GEV
Lim [25]	2017	Korea	64	100	Cirrhosis + HVPG ≥ 12 mmHg
Kimer [24]	2017	Denmark	54	27.8	Cirrhosis + ascites + HVPG ≥ 10 mmHg

^a^Gastroesophageal varices

^b^Hepatic venous pressure gradient

^c^Child-Pugh.

A study [21] had three arms, and the antibiotic and probiotic treatment arms were included as intervention groups. We named them Gupta (R) and Gupta (P) respectively, compared with the third arm that used placebos (control group). Hence, there were seven comparison groups in our analysis. Two groups [21, 22] evaluated probiotics in the management of cirrhosis while five [20, 21, 23–25] groups evaluated antibiotics in the management of cirrhosis. Regrettably, no studies that used prebiotics or fecal bacteria transplantation for treatment fulfilled the inclusion criteria for our analysis. All the studies assessed PH though HVPG measurement, four [22–25] studies required patients should diagnosed with CSPH(HVPG>10 mmHg, even more>12mm Hg); gastroesophageal varices were included in the remaining studies [20, 21]. Table 2 summarizes the type, dose and period of MTT. The duration varied from 28 to 90 days.

**Table 2:** The characteristics of patients in the intervention and control group (means ± standard deviation).

Author	Arm	Treatment schedule and dose	Days	No. of patients	Males (%)	Age	Child-Pugh score		MELD score
Albillos [20]	Treatment	Norfloxacin 400mg bid	28	12	NR^a^	NR		NR	NR
	Control	Placebo	28	6	NR	NR		NR	NR
Kemp [23]	Treatment	Norfloxacin 400mg bid	28	8	56.3	59±2.4		6.3±0.3	8.7±1.1
	Control	Placebo	28	8	56.3	59±2.4		6.3±0.3	6.3±0.3
Jayakumar [22]	Treatment	VSL#3 (3600 billion CFU)	56	7	71.4	50±8.9		8±1.5	11±5.2
	Control	placebo	56	8	87.5	53.5±3.7		8.5±1.5	13.5±3.6
Gupta [21]	Treatment	Norfloxacin 400mg bid+ Propranolol	60	31	77.0	42±14		8±2	13.3±4
	Treatment	VSL#3^b^ (900 Billion CFU^c^) + Propranolol	60	31	84.0	43±11		9±2	15.1±5
	Control	Propranolol	60	32	63.0	45±11		8±2	13.8±4
Lim [25]	Treatment	Rifaximin 1200mg/day+ Propranolol	90	16	93.8	51.2±9.6		6.9±2.2	10.1±3.9
	Control	Propranolol	90	48	85.4	48.8±9.7		7.1±1.7	11.4±4.1
Kimer [24]	Treatment	Rifaximin 550mg bid	28	36	86.1	58.5±8.8		8.6±1.3	12.5±4.3
	Control	Placebo	28	18	77.8	52.5±10		7.8±0.9	9.9±2.4

^a^No record

^b^A combination of viable lyophilized bacteria

^c^Clonal formation unit

### Quality of the included studies

**Fig 2:**
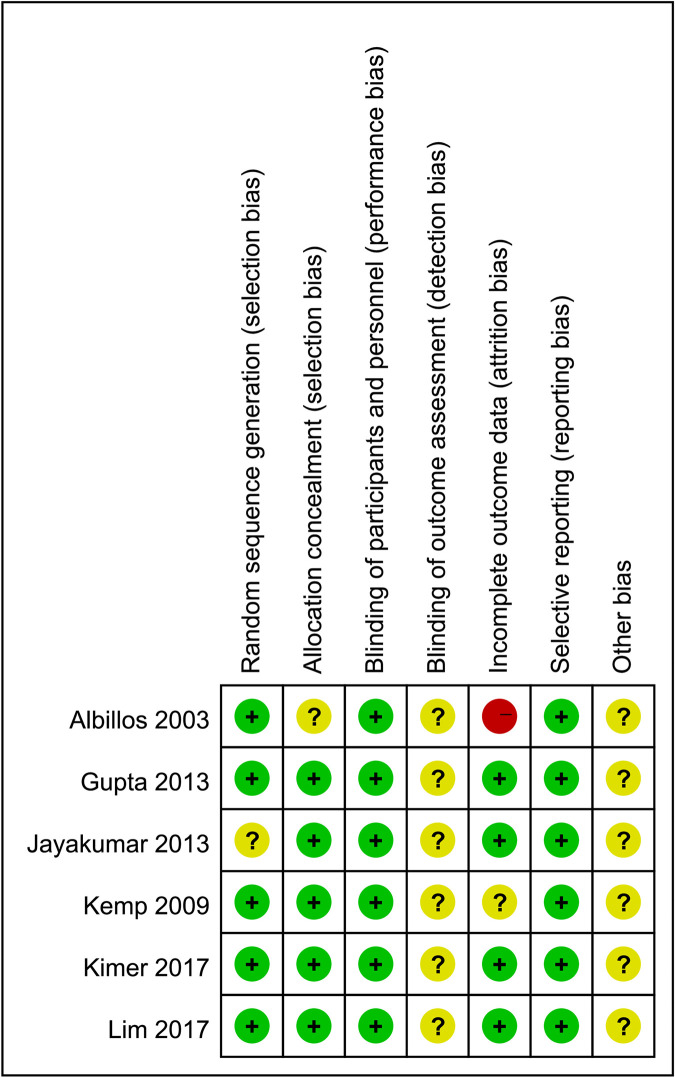
Quality assessment of RCTs for risk of bias.

Overall, the majority [20, 21, 23–25] of the included studies (83.3%) showed adequate randomization. One trial [24] adopted the random number table method to divide the groups, and four trials [20, 21, 23, 25] randomized participants by a computer‐generated randomization list. Allocation concealment was considered low risk for five studies [21–25] (83.3%), but unclear for one [20], due to insufficient detail. Blinding of investigators and participants was performed in all the studies. Thus, all studies had a low risk of bias regarding blinding. All the studies reported prespecified outcome measurements. We judged one study [20] as having a high risk of bias, as it did not provide a clear explanation of withdrawals or dropouts. The risk of bias assessment is summarized in Fig 2.

### Outcomes

#### HVPG

All six trials evaluated the variation in HVPG treated MTT. Compared with the control groups, six groups showed that MTTs achieved a reduction in HVPG. However, statistical significance was found in only one group [25] (P = 0.034).

**Fig 3:**
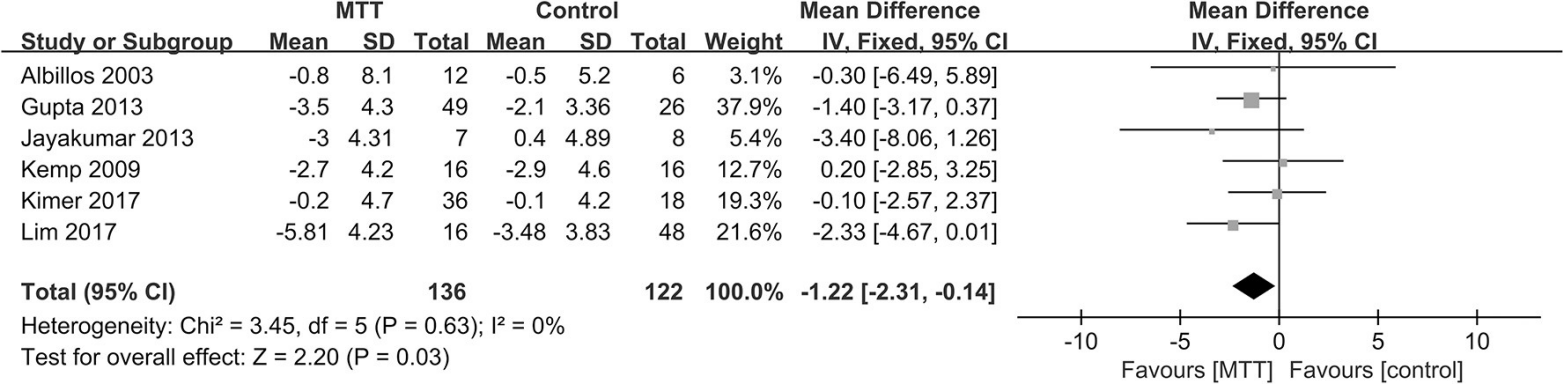
Forest plot of MTT on HVPG reduction in cirrhosis patients.

Altogether, in the meta-analysis (Fig 3), no heterogeneity was identified (P = 0.63, I^2^ = 0%), and we employed a fixed effect model. Antibiotics and probiotics were associated with a reduction in HVPG (-1.22 mmHg, 95% CI: -2.31, -0.14 mmHg; P = 0.02)., which may indicate a HVPG decrease with MTTs.

**Fig 4:**
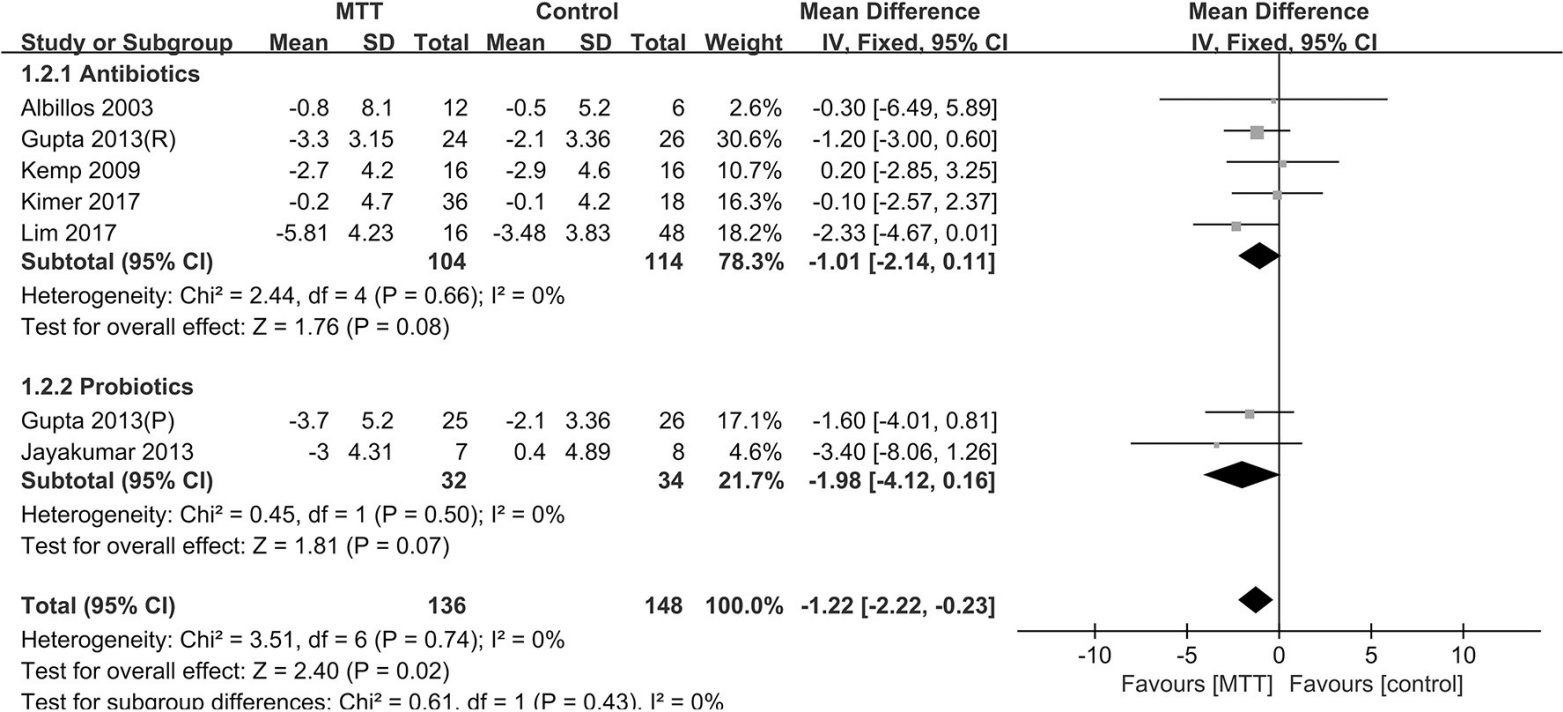
Forest plot of antibiotics and probiotics on HVPG reduction in cirrhosis patients.

Subgroup analysis was carried out based on the type of MTT to compare the different efficiencies between the antibiotic and probiotic groups (Fig 4). In the subgroup analysis, the effect was consistent despite different types being loaded. The probiotic group did not show a greater HVPG reduction (-1.98 mm Hg, 95% CI: -4.12, 0.16; P = 0.07) than the antibiotics group (-1.01 mm Hg, 95% CI: -2.14, 0.11 mmHg; P = 0.08), without revealing statistical significance. Perhaps this difference may become statistically significant as the sample size increases.

**Fig 5:**
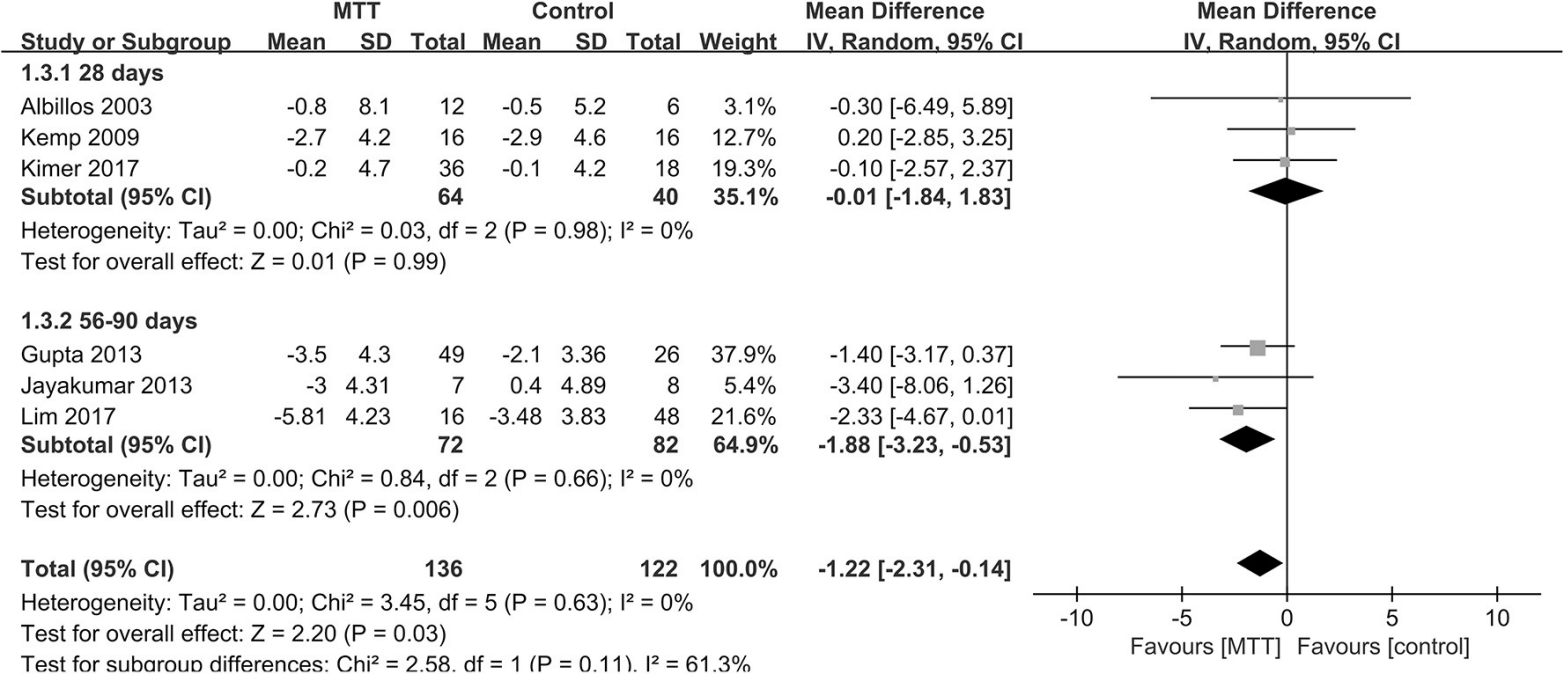
Forest plot according to duration of MTT on HVPG reduction in cirrhosis patients.

Subgroup analysis was also carried out in accordance with the period of therapy (Fig 5). In the subgroup analysis, the effect was different between the two groups (P = 0.03). The longer duration group showed an HVPG reduction of -1.88 mmHg (95%CI: -3.23, -0.53; P = 0.006), which indicated a significant reduction in longer therapy. The subgroup analysis did not show heterogeneity (P = 0.66, I^2^ = 0%).

#### Second outcomes

Three groups [20, 24, 25] evaluated the effects of serum levels of LBP. Compared with the control groups, all showed that MTTs achieved a reduction in serum levels of LBP (P = 0.018, P = 0.002, P<0.01). Five groups [20–22, 25] evaluated the effect on serum levels of IL-6. Only two [20, 24] showed statistical significance in the MTT groups (P<0.01, P = 0.026). Serum concentrations of NO decreased markedly in the MTT group in one [20] of the four groups (P<0.05).

**Fig 6:**
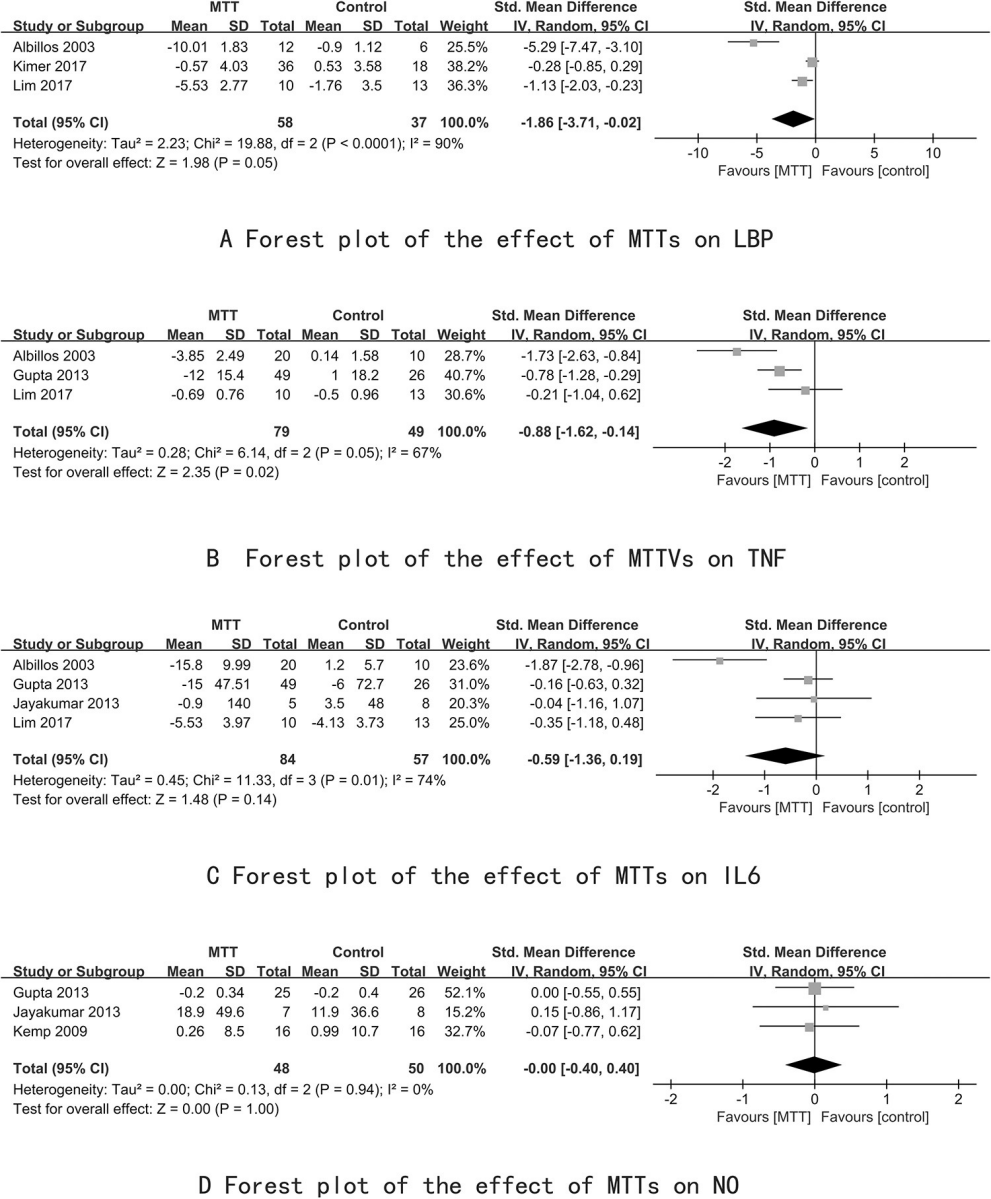
Forest plot of MTT on LBP, TNF-α, IL-6 and NO.

In meta-analyses, the SMD of LBP level was -1.86 (95% CI:-3.71, 0.02;P = 0.05), reducing in MTT, but accompanied by high heterogeneity (P<0.0001, I^2^ = 90%) (Fig 6A). The TNF-α level (SMD: -0.88; 95% CI: -1.62, -0.14; P = 0.02) was reduced significantly, followed by high heterogeneity (P = 0.05, I^2^ = 67%) (Fig 6B). The MD of the level of IL-6 was -0.59 (95% CI: -1.36, 0.19; P = 0.14), indicating a nonsignificant decrease with MTT and with moderate heterogeneity (P = 0.01, I^2^ = 74%) (Fig 6C). For the serum NO level, no heterogeneity was identified(P = 0.94, I^2^ = 0%) and did not decrease significantly (SMD: -0.00; 95% CI: -0.40, 0.40; P = 1.00) (Fig 6D).

#### Trial sequential analysis

**Fig 7:**
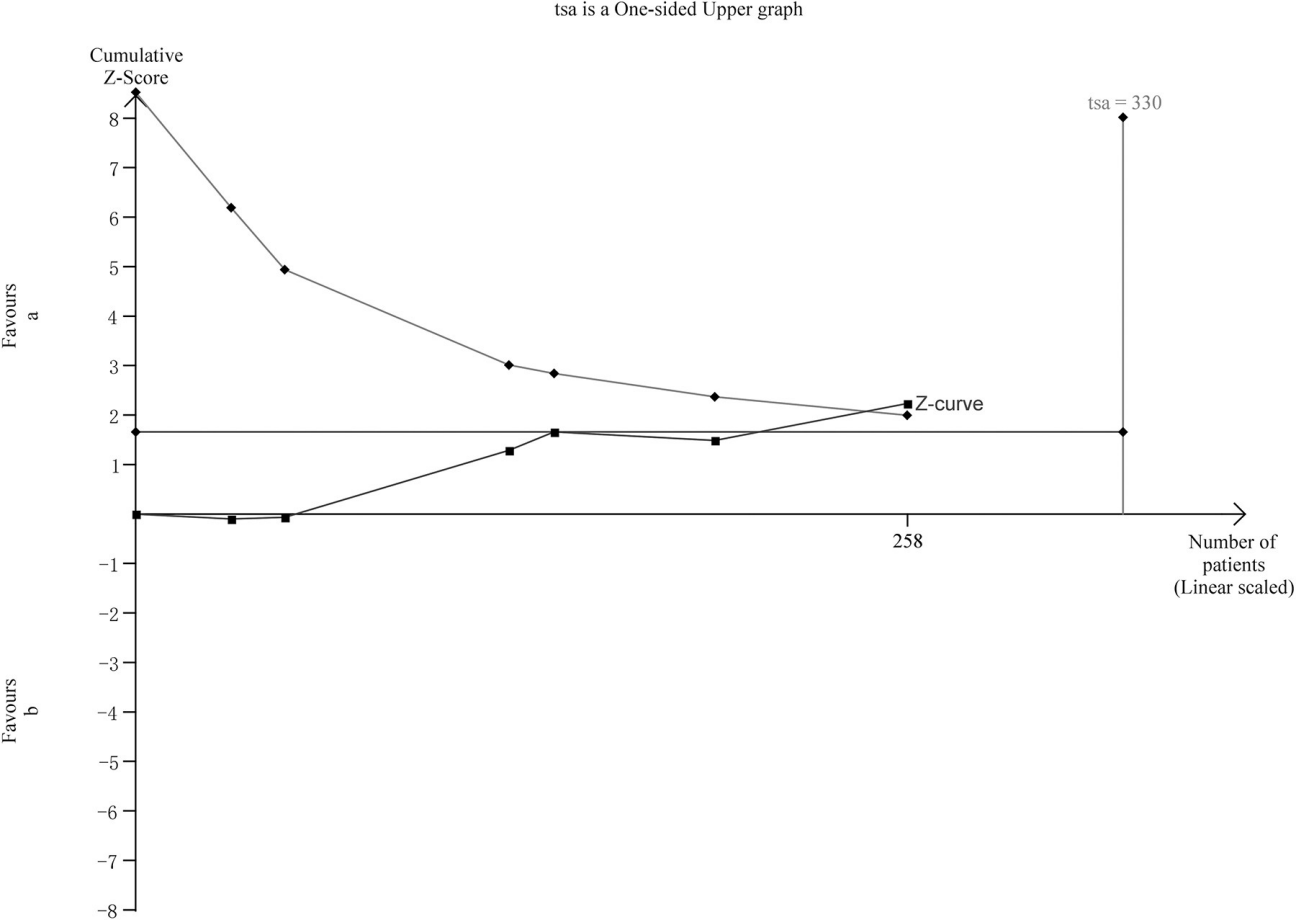
Trial sequential analysis.

In the TSA of HVPG reduction (Fig 7)., the required information size is 330. The cumulative Z curve crossed the trial sequential monitoring boundaries without the achievement of RIS, we think the anticipated intervention effect may have been reached.

## Discussion

Given numerous clinical studies that identify the association between the gut microbiome and PH, PH is aggravated by hyperdynamic circulation and proinflammatory and profibrotic stimuli caused by BT [13]. Treatment targeting the gut-liver axis via modification of microbiota composition and proinflammatory have attracted increasing interest [26]. In our meta-analysis, we comprehensively conducted an assessment of the effects of MTTs on HVPG and BT-related outcomes. The results of our meta-analysis indicate that MTTs may have beneficial effects on reducing HVPG in patients with cirrhosis and portal hypertension. This reduction in HVPG might have a positive clinical impact. Similar to the study suggestions, increasing HVPG is followed by a higher risk of decompensation or death [27]. However, reducing 1–2 mmHg of portal pressure may not significantly alter the clinical outcome. Still, it underlined the potential utility of these therapies and should trigger more concerns.

When the analysis was stratified according to the type of MTT, probiotics did not reduce HVPG in cirrhosis more efficiently than antibiotic therapy, because the differences were not statistically significant. Considering the advanced effects and resistance to the long-term antibiotic therapy [28], probiotics in cirrhosis may show more potential for clinical application with more needed validation.

Concerning the period of therapy, a longer course identified greater reduction in HVPG, suggesting that more time is required to observe the effect of MTT. This is consistent with the result showing that the chronic use of antibiotics markedly relieved the risk of complications related to PH and improved overall survival [29]. However, due to insufficient information and the various definitions of adherence, we were not able to clarify the adverse reactions in the long term.

In addition, we also estimated the nonhemodynamic effects mentioned above. Compared with the control group, LPB and TNF-α were reduced after MTT. Even with the small sample, these consequences were remarkable. Microbial products activate TLRs, which can activate to produce proinflammatory cytokines and extracellular matrix proteins, ultimately leading to hepatic fibrosis [30].

We hypothesized that MTTs affect the intestinal microbiota composition and then decrease BT, leading to endotoxemia reduction. Unfortunately, we did not further identify whether BT-related markers and proinflammatory cytokines significantly declined in the group of hemodynamic responders with MTT. This could prove our hypothesis that BT induces an inflammatory response and then exacerbates liver tissue injury and fibrosis progression, which ultimately increases portal pressure. Thus, exploring the effect of the HVPG response with MTT would be a direction for future research.

There are certain limitations in our meta-analysis. First, the number of included studies was limited, with a limited sample size. In addition, no RCTs using prebiotics or fecal bacteria transplantation for treatment fulfilled the inclusion criteria in our analysis, and most of the data coming from studies on antibiotics. Moreover, the patient population differed from study to study, and we were not able to assess the underlying possible effects of MTTs in decompensated or compensated patients. Last, it was uncertain whether such low reduction in HVPG could alter the clinical outcome.

In conclusion, our results show that microbiome-targeted therapies may reduce portal pressure in cirrhosis patients with portal hypertension. Subgroup analysis shows that with increasing duration, HVPG decreases more. At the same time, the serum levels of LBP and TNF-α, as BT markers, were also reduced after MTT. Some limitations are mentioned above, but we still believe our results are inspiring and worth conducing larger trials to confirm MTTs as treatments for PH, particularly given the cost- effectiveness of these treatments.

In summary, modulation of the gut microbiota through probiotics or antibiotics is considered a promising therapeutic strategy for PH. Additional research involving more cirrhosis and PH patients is required to explore the efficacy of MTTs.

## Conclusion

The result of this mate-analysis of RCTs, demonstrated that MTTs may be associated with reduction on HVPG in patients with cirrhosis and PH. Microbiome-targeted therapies merit additional large-sample studies to define the efficacy in HVPG. All data underlying this finding is fully available in S3 File.

## Supporting information

S1 File PRISMA 2009 checklist. (DOC)

S2 File The full electronic search strategy. (DOCX)

S3 File Data set. (XLSX)
