# Supplementary material for: Antibiotics and probiotics on hepatic venous pressure gradient in cirrhosis: A systematic review and a meta-analysis
Source: PLoS One. 2022 Aug 30;17(8):e0273231. doi: 10.1371/journal.pone.0273231 (PMC9426916; doi:10.1371/journal.pone.0273231)
Supplement: S2 File — (DOCX) [file pone.0273231.s002.docx]

PubMed

#1 ((("Liver Cirrhosis"[Mesh]) OR ("Liver Cirrhosis"[Mesh])) OR ("Hepatic Cirrhosis"[Title/Abstract])) OR (Cirrhosis [Title/Abstract])

#2 (("Hypertension, Portal"[Mesh]) OR ("Portal Hypertension"[Title/Abstract])) OR ("Venous Pressure"[Title/Abstract])

#3 (((("Anti-Bacterial Agents"[Mesh]) OR ("Anti-Bacterial Agents"[Title/Abstract])) OR ("Anti-Bacterial Agents"[Title/Abstract])) OR (Antibiotic [Title/Abstract])) OR (Bacteriocides[Title/Abstract])

#4 ((("Rifaximin"[Mesh]) OR ("Rifaximin"[Title/Abstract])) OR (Xifaxan[Title/Abstract])) OR (L105[Title/Abstract])

#5 (((("Neomycin"[Mesh]) OR (Neomycin [Title/Abstract])) OR ("Norfloxacin"[Mesh])) OR ("Norfloxacin"[Title/Abstract])) OR (Noroxin[Title/Abstract])

#6 ((((((((((((((("Probiotics"[Mesh]) OR (Probiotics[Title/Abstract])) OR (Probio*[Title/Abstract])) OR ("Lactobacillus"[Mesh])) OR (Lactobacillus[Title/Abstract])) OR (Lactobacilli[Title/Abstract])) OR (Acidophilus[Title/Abstract])) OR (Bifidobacterium[Title/Abstract])) OR (Bifido[Title/Abstract])) OR (Enterococcus[Title/Abstract])) OR (Enterococci[Title/Abstract])) OR ("Escherichia coli"[Title/Abstract])) OR (Saccharomyces[Title/Abstract])) OR (Bacillus[Title/Abstract])) OR (VSL#3[Title/Abstract])) OR (Boulardii[Title/Abstract])

#7 ((((((((((("Prebiotics"[Mesh]) OR (Prebiotics [Title/Abstract])) OR (Prebio*[Title/Abstract])) OR (Fructan*[Title/Abstract])) OR (Oligofructose [Title/Abstract])) OR (Fructo*[Title/Abstract])) OR (Fructooligosaccharide[Title/Abstract])) OR (Fructo-oligosaccharide[Title/Abstract])) OR (Xylose[Title/Abstract])) OR (oligosaccharide[Title/Abstract])) OR (Inulin[Title/Abstract])) OR (Lactulose[Title/Abstract])

#8 (("Synbiotics"[Mesh]) OR (Synbiotics [Title/Abstract])) OR (Synbio*[Title/Abstract])

#9 (("Fecal Microbiota Transplantation"[Mesh]) OR ("Fecal Microbiota Transplantation"[Title/Abstract])) OR (FMT[Title/Abstract])) OR (((Microbiota [Title/Abstract]) OR (microbiome [Title/Abstract]))

#10 #3 OR #4 OR #5 OR #6 OR #7 OR #8 OR #9

#11 #1 AND #2 AND #10

Cochrane

#1 MeSH descriptor: [Liver Cirrhosis] explode all trees

#2 (“hepatic cirrhosis”):ti,ab,kw OR (cirrhosis):ti,ab,kw

#3 #1 OR #2

#4 MeSH descriptor: [Hypertension, Portal] explode all trees

#5 ("Portal hypertension"):ti,ab,kw OR ("Venous Pressure") OR (HVPG) :ti,ab,kw

#6 #4 OR #5

#7 MeSH descriptor: [Probiotics] explode all trees

#8 MeSH descriptor: [Anti-Bacterial Agents] explode all trees

#9 MeSH descriptor: [Prebiotics] explode all trees

#10 MeSH descriptor: [Synbiotics] explode all trees

#11 MeSH descriptor: [Fecal Microbiota Transplantation] explode all trees

#12 (Antibiotic):ti,ab,kw OR (Bacteriocides):ti,ab,kw

#13 #12 or #8

#14 (Rifaximin):ti,ab,kw OR (Neomycin):ti,ab,kw OR (Norfloxacin):ti,ab,kw

#15 #13 or #14

#16 (Probio*):ti,ab,kw OR (Lactobacillus):ti,ab,kw OR (Lactobacilli):ti,ab,kw OR (Acidophilus):ti,ab,kw OR (Bifidobacterium):ti,ab,kw

#17 (Bifido):ti,ab,kw OR (Enterococcus):ti,ab,kw OR (Enterococci):ti,ab,kw OR ("Escherichia coli"):ti,ab,kw OR (Saccharomyces):ti,ab,kw

#18 (Bacillus):ti,ab,kw OR ("VSL"):ti,ab,kw OR (Boulardii):ti,ab,kw

#19 #7 OR #16 OR #17 OR #18

#20 (Prebio*):ti,ab,kw OR (Fructan*):ti,ab,kw OR (Oligofructose):ti,ab,kw OR (Fructo*):ti,ab,kw

#21 (Fructo-oligosaccharide):ti,ab,kw OR (Xylose):ti,ab,kw OR(oligosaccharide):ti,ab,kw OR (Inulin):ti,ab,kw OR (lactulose):ti,ab,kw

#22 #9 OR #20 OR #21

#23 ("Fecal Microbiota Transplantation"):ti,ab,kw OR (FMT) :ti,ab,kw OR (Microbiota Transplantat*):ti,ab,kw

#24 #15 OR #19 OR #22 OR #23

#25 #3 AND #6 AND #24

Web of Science

#1 ("Liver Cirrhosis") OR ("hepatic cirrhosis ") OR (cirrhosis)

#2 ("Portal hypertension") OR ("Venous Pressure") OR (HVPG)

#3 (Antibiotics) OR (Bacteriocides) OR ("Anti-Bacterial Agents") OR (Rifaximin) OR (Neomycin) OR (Norfloxacin)

#4 (Probiotics) OR (Probio*) OR (Lactobacillus) OR (Lactobacilli) OR (Acidophilus) OR (Bifidobacterium) OR (Bifido) OR (Enterococcus) OR (Enterococci) OR ("Escherichia coli") OR (Saccharomyces) OR (Bacillus) OR (VSL) OR (Boulardii)

#5 (Prebiotics) OR (Prebio*) OR (Fructan*) OR (Oligofructose) OR (Fructo*) OR (Fructooligosaccharide) OR (Fructo-oligosaccharide) OR (Xylose) OR (oligosaccharide) OR (Inulin) OR (lactulose)

#6 (Synbiotics) OR (Synbio*)

#7 ("Fecal Microbiota Transplantation") OR (FMT) OR (Microbiota Transplantat*)

#8 #3 OR #4 OR #5 OR #6 OR #7

#9 #1 AND #2 AND #8

Embase

#1 ‘liver cirrhosis‘/exp

#2 ‘hepatic cirrhosis ‘:ti,ab,kw OR cirrhosis:ti,ab,kw

#3 #1 OR #2

#4 ‘portal hypertension’/exp

#5 ‘venous pressure’: ti,ab,kw OR ‘portal hypertension’ :ti,ab,kw

#6 #4 OR #5

#7 ‘antibiotic agent’/exp

#8 antibiotic: ti,ab,kw OR bacteriocides :ti,ab,kw OR rifaximin: ti,ab,kw OR neomycin: ti,ab,kw OR norfloxacin: ti,ab,kw

#9 #7 OR #8

#10 ‘probiotic agaent‘/exp

#11 Probiotics: ti,ab,kw OR Probio*: ti,ab,kw OR Lactobacillus: ti,ab,kw OR Lactobacilli: ti,ab,kw OR Acidophilus: ti,ab,kw OR Bifidobacterium: ti,ab,kw OR Bifido: ti,ab,kw OR Enterococcus: ti,ab,kw OR Enterococci: ti,ab,kw OR ‘Escherichia coli’ : ti,ab,kw OR Saccharomyces: ti,ab,kw OR Bacillus: ti,ab,kw OR Boulardii: ti,ab,kw

#12 #10 OR #11

#13 ‘prebiotic agaent’/exp

#14 prebiotics: ti,ab,kw OR Prebio*: ti,ab,kw OR Fructan* : ti,ab,kw OR Oligofructose: ti,ab,kw OR Fructo* : ti,ab,kw OR Fructooligosaccharide : ti,ab,kw OR Fructooligosaccharide : ti,ab,kw OR Xylose: ti,ab,kw OR oligosaccharide: ti,ab,kw OR Inulin: ti,ab,kw OR lactulose: ti,ab,kw

#15 #13 OR #14

#16 ‘synbiotic agaent/’exp

#17 Synbiotics OR Synbio*: ti,ab,kw

#18 #16 OR #17

#19 ‘fecal microbiota transplantation‘/exp

#20 #9 OR #12 OR #15 OR #18 OR #26

#21 #3 AND #6 AND #20
